# Risks for eating disorder in ultra-endurance athletes and the role of training volume: a cross-sectional study

**DOI:** 10.3389/fspor.2025.1708869

**Published:** 2025-12-10

**Authors:** Jill Colangelo, Alexander Smith, Norman Bitterlich, Ana Buadze, Michael Liebrenz

**Affiliations:** 1 Department of Forensic Psychiatry, University of Bern, Bern, Switzerland; 2 Faculty of Sport, Technology, and Health Sciences, St. Mary’s University Twickenham, London, United Kingdom; 3 Independent Researcher, Chemnitz, Germany; 4 Department of Psychiatry, Psychotherapy and Psychosomatics, Psychiatric Hospital, University of Zurich, Zurich, Switzerland

**Keywords:** REDs, ultra endurance, sports psychiatry, mental health, disordered eating, training volume

## Abstract

**Background:**

Characterized by prolonged physical activity, ultra-endurance sports (UES) have attracted increasing popularity. Though moderate exercise can confer mental health benefits, the effects of extreme training volumes remain unclear. Instead, emerging research suggests a link between high training loads and increased psychiatric vulnerabilities, including for eating disorders (ED). Consequently, this study aimed to investigate trends in ED risk in a sample of ultra-endurance athletes (UEA) and the relationship to training volumes.

**Methods:**

A cross-sectional survey was administered among UEA using the Eating Attitudes Test-26 (EAT-26), alongside gathering demographic data and information on training hours. Athletes were categorized into three training volume groups: <10 h, 10–20 h, and >20 h per week. Statistical tests, including ANOVA, Mann–Whitney *U*-tests, and Receiver Operating Characteristic analysis, were used to examine correlations between training volume and ED risk while adjusting for age and gender.

**Results:**

Of the *n* = 531 participants, 11% (*n* = 58) scored above the clinical threshold on the EAT-26, indicating a need for further evaluation. Equally, 20.3% (*n* = 107) exhibited at least one conspicuous behavioral symptom of disordered eating. Training volume was a significant predictor of ED risk (F = 31.494, *p* < 0.001), with athletes training more per week exhibiting higher EAT-26 scores and greater engagement in disordered eating behaviors. Specifically, ROC analysis identified 14 h per week as the threshold for heightened ED susceptibility, with a sensitivity of 53.4% and specificity of 76.1%.

**Conclusion:**

There was a significant association between high training volumes and increased ED risk among this UEA sample, suggesting a need for greater awareness of psychiatric vulnerabilities in endurance athletes. Given the growing popularity of UES, better education on the risks associated with high training volumes may be necessary to promote healthier training practices, underpinned by further research into its physiological and psychological consequences.

## 1 Introduction

Interest and participation in ultra endurance sports (UES) has grown, influenced by media coverage, sponsorships, and sociocultural enthusiasm for fitness, amongst other factors (1, 2). While definitions vary, UES encompasses events over six hours in duration [e.g., ultra marathon, ironman-distance triathlon (e.g., Ironman® branded events), ultra distance cycling, ultra swimming, etc.], which often necessitate prolonged training periods (1). Despite their burgeoning popularity, research on the psychopathological implications of UES remains limited (3). Typically, sporting participation can confer positive psychological effects and is recurrently incorporated in mental health promotion schemes and therapeutic programmes (4). Nevertheless, athletes may also exhibit mental illness risks at equivalent risks to the general population (5). While moderate exercise can have an advantageous impact on mental health and wellbeing (6, 7), it is unknown whether this extends to the often substantial volumes of physical activity inherent in UES. In this regard, surprisingly few investigations have explored whether mental health benefits increase with higher physical activity or if there is a U-shaped relationship meaning these may peak, then decline after a saturation point (8). In the limited research available, a trend toward decreasing mental health with increasing volumes of physical activity has been observed (5), as have heightened psychiatric vulnerabilities across UES communities (3).

Sizeable rates of eating disorders (ED) and associated risk factors have been identified among UEA. Among 583 triathletes, 28% of female and 11% of male participants were found to be at-risk for ED. Elsewhere, among 1,445 male and female ultrarunners, 43% may have been engaging in disordered eating habits (9, 10). Correspondingly, other studies among 123 ultrarunners found that 44.5% of male and 62.5% of athletes exhibited sizeable risks for disordered eating (11). More generally, anorexia nervosa and bulimia nervosa have both been detected in athletes across various ages and genders, as well as in elite and amateur participation levels (12–14).

Specifically, exercise bulimia, in which a person purges by burning calories through excessive exercise, has also been included in similar discussions about athletes (15). Binge eating disorder, the most prevalent ED, may be common in sporting settings due to weight/body composition pressures, caloric restriction, and a perception of performance gains with lower BMI (16). Notably, ED have been associated with large amounts of physical activity in both sport and non-sporting contexts (17, 18). UES may incur additional strain on athletes due to its defining feature of large volumes of physical activity, which can have unique consequences for mental health, nutrition, preparation, external stressors, and fueling to support participation (19). Equally, unlike other sports where volumes of exercise are commensurate with expertise and/or elite status (20), in UES, athletes of all levels are required to train and/or compete with high volumes of physical activity (1). Therefore, if indicators of mental disorders are prevalent in UES, it would affect a larger population than just elites and/or professional athletes.

Furthermore, given that UES requires a high level of physical activity, it is critical to understand if participation in these sports is associated with higher ED risk. Nutrition and fueling are integral components of UES and may contribute to heightened vulnerabilities, such as fatigue, difficulties with thermal regulation, and somatic injuries (21). Though proper nutrition is important for all physical activity, it can be a critical factor in UES; both health and performance decrements have been highlighted among underfueled athletes (21–23). The increased duration of activities entails high energy expenditure, which must be managed through nutritional intake during exercise, as well as after training and/or racing efforts (24). UEA can expend thousands of calories over a single, long-distance effort (21). Moreover, environmental variations like temperature and terrain may alter individual needs and how nutrition must be taken (i.e., swimmers in water vs. cyclists riding a bicycle) (21). As gastrointestinal issues are also frequently identified in UEA, special attention is often needed to take in well-tolerated and effective energy sources (24). Likewise, UEA can demonstrate weight and body image dissatisfaction and subsequently restrict calories (9), which may contribute to the development of ED (25).

Although the concepts of exercise addiction and exercise dependence (EXD) have been investigated in disparate contexts, they have not yet been officially recognized as distinct clinical diagnoses in the International Classification of Diseases, 11th Revision (ICD-11)_ or the Diagnostic and Statistical Manual of Mental Health Disorders, 5th Edition (DSM-5). However, their potential impact on athletes, particularly in relation to ED, remains a subject of ongoing research (26). ED and EXD can co-occur in athletes, though not all those who experience exercise dependence will have ED (and vice versa) (26). *Secondary* EXD is characterized by physical activity that is compensatory for weight and/or body image concerns, whereas *primary* EXD is typified by an intrinsic motivation and enthusiasm for training (27). In UEA, excessive physical activity can induce EXD (28), and though the term “excessive” may be subjective, even normal UES training could invoke concerns about over-exercise due to the volume necessary for adequate training (19). Concomitantly, since UEA may be at-risk for EXD, they may also have heightened ED vulnerabilities (29). Furthermore, Relative Energy Deficiency in Sport (REDs), a condition affecting multiple body systems resulting from inadequate caloric intake (Low Energy Availability- LEA), is a concern for UE athletes (10, 30). REDs can affect athletes of any gender, whether caloric restriction is intentional or not, and has been observed in amateur and elite athletes (30, 31).

Disordered eating behaviors may include atypical presentations of ED (32) and/or attitudes and habits around food and eating that are irrational or illogical (33). Disordered eating does not always lead to ED but it can cause distress and impairments to everyday functioning, alongside long-term mental health issues (33, 34). Disordered eating behaviors can be particularly problematic when they are culturally accepted because “normal eating” is overlooked in the context of sociocultural and community-based conventions (35). Such behaviors may be encouraged by media and in-groups, especially where the resulting body composition changes or other outcomes may be deemed favorable (35). Evidence indicates that UEA may engage in restrictive eating (36) and that underfueling is a prominent phenomenon (21). Anecdotally, UEA have shared their experiences with ED and disordered eating in elite and amateur level contexts (37, 38).

Given the complexities of UES, the unique contribution of high volume physical activity, and the lack of available data in this area, this research was aimed at examining trends that would relate to ED risk in a sample of UEA. Owing to the distinctively high training volume of UES, analysis also centred around the number of hours per week that athletes engaged in physical activity and whether that affects ED or disordered eating trends.

## 2 Methods

### 2.1 Study design

This study used a cross-sectional design based on self-report psychometric instruments. The online survey was available through the Qualtrics platform and consisted of eleven demographic questions (see appendix), the Patient Health Questionnaire (PHQ), and the Eating Attitudes Test-26 (EAT-26). The current research is based on a master's degree thesis project designed to better understand general trends in mental health, risks for mental disorder including depression, and training volume in UEA (39).

### 2.2 Participant recruitment

Recruitment advertisements were placed on several websites and media outlets popular with the UES community (e.g., irunfar.com and Ultrarunning Magazine). Subjects were also recruited from the Fast Women newsletter, and posts were made on large trail running, triathlon, and ultracycling groups on Facebook. The advertisement explicitly stated that participants would be answering questions about mental health in ultra endurance athletes. Inclusion criteria were; people over the age of 18 and self-identification as UEA. All research was carried out in accordance with guidelines on human subjects research set forth by Harvard University Internal Review Board (approval IRB20-1361). Informed consent was obtained from all participants. As no participant was below the age of consent, no guardians signed consent forms. The survey was available for one month between September 10-October 10, 2020; all data was collected during this time period. As participation was anonymous, the geographical location of participants is unknown, though the recruitment advertisements were all disseminated via North American-based outlets.

### 2.3 Materials and measures

#### 2.3.1 Demographic questionnaire

The survey collected demographic data, including age, gender, and race; the latter classifications were based on US Census categories (40). Athletes were asked about the sports they competed in, whether they had participated in an event in the last twelve months, and whether they planned to compete in a UES event in the upcoming twelve months. Additionally, respondents reported their weekly training hours, with “training” explicitly defined as all physical activity related to their sport, including cross-training (e.g., 10 h of running as a primary sport plus 2 h of rock climbing as cross-training equals 12 total training hours per week). As definitions and conceptualisations can vary (19), the number of weekly training hours represents “training volume” within this study. The survey also assessed if athletes had ever received a mental health diagnosis and whether that diagnosis was received before or during their UE sport participation. Finally, respondents were asked if they were in recovery from substance abuse.

#### 2.3.2 Eating attitudes test-26 (EAT-26)

This study encompassed all questions from the EAT-26 and the PHQ. The present analysis solely focuses on results from EAT-26, which has been shown validity and reliability for evaluating self-reported ED (*r* = 0.87, *p* < 0.01) such that those recovered from ED score lower (41). Specifically, the EAT-26 provides a clearer distinction between anorexia nervosa and bulimia nervosa (42). The test can also be used to uncover binge eating disorder since it separates bingeing from purging behaviors (42), and has previously been used in studies of UEA (9, 43).

The self-assessment contains twenty-six questions about attitudes toward eating which are answered on a six-point Likert scale. Answers are designed to assess frequency using the phrases, “never” (0), “rarely” (0), “sometimes” (0), “often” (3), “usually” (3), and “always” (3). Those who score twenty or more on the EAT-26 should have a follow-up with a mental health specialist and scores less than twenty may also indicate the presence of abnormal eating behaviors. Correspondingly, scores over twenty do not suggest certainty of the presence of ED.

The EAT-26 also contains a bank of five behavioral questions (A-E) which ascertain whether an individual has engaged in actions that are associated with disordered eating in the prior six months. These ask about binge eating (A), self-induced vomiting (B), laxative, diet pill, and diuretic use (C), exercising for weight control (D), and if respondents had weight loss of 20 pounds or more (E). For items A-D, possible responses are “never” (0), “once a month or less” (0), “2–3 times per month” (1), “once a week” (1), “2–6 times per day” (1), and “once a day or more” (1). Item E includes “no” (0) or “yes” (1) responses. If a subject selects at least “once a month or less” on B and C, “2–3 times per month” on A, “once a day or more” for D, or “yes” on E, it is suggested that they seek evaluation by a specialist irrespective of their EAT score (these are hereafter referred to as conspicuous behaviors).

### 2.4 Procedure

Prior to the study, the research design (IRB20-1361) was approved by the Internal Review Board at Harvard University. Participants followed a link to the Qualtrics platform where they were informed that the study would cover mental health in ultra endurance sport and that their participation would be anonymous. They answered three screening questions and read and signed a consent form for participation. It was possible to exit the study at any time. Participants answered eleven demographic questions, the PHQ, and finally, the EAT-26. No reward, lottery, or compensation was offered for participation.

### 2.5 Data analysis

All data collected via the Qualtrics survey platform were exported and uploaded into IBM SPSS Statistics (Version 26, IBM Corp.) for analysis. Descriptive statistics were calculated for demographic data, including means, standard deviations, and median and 1st/3rd quartile frequencies, to summarize participant characteristics. Subgroup differences in continuous data, such as EAT-26 scores across training volume groups, were tested using the Mann–Whitney *U*-test, given the non-parametric nature of the data. For proportions, such as the frequency of disordered eating behaviors between training volume groups, Chi-square (*χ*^2^) tests were applied to assess significant associations.

A one-way analysis of variance (ANOVA) supplemented by correlation analysis was conducted to assess the relationship between training volume and overall EAT-26 scores. *post-hoc* comparisons were performed using Tukey's HSD test to identify significant differences between groups. Where appropriate, multivariate analyses adjusted for age and gender were conducted using an analysis of covariance (ANCOVA) to evaluate the effects of these variables on training volume and EAT-26 scores. To identify the most selective threshold of training volume associated with heightened risk for ED, Receiver Operating Characteristic (ROC) analysis was performed to calculate sensitivity, specificity, and the Youden Index [% = sensitivity [%] + specificity [%]−100%]. This enabled the determination of a training volume threshold predictive of elevated EAT-26 scores.

All tests were two-tailed, and statistical significance was set at *p* < 0.05. Results are reported with corresponding test statistics (e.g., *U* values for Mann–Whitney, *χ*^2^ values for Chi-square, and *F* values for ANOVA/ANCOVA) and significance levels. Unless otherwise stated, effects of multiple testing are ignored in the exploratory analysis.

## 3 Results

### 3.1 Sample overview

645 participants responded to the survey, of which, *n* = 531 records were complete and eligible for analysis; 59.3% (315) respondents were male and 40.7% (216) were female. In one case, the gender was noted as “male.female” but in a later question they self-reported as female and were therefore categorized as female.

All participants were self-described UEA between 19 and 73 years old (*n* = 156 (29.4%) < 36 years old, *n* = 197 (37.1%) 36–45 years old, *n* = 125 (23.5%) 46–55 years old, and *n* = 53 (10.0%) ≥56 years old). Athletes engaged in sports such as ultrarunning, trail running, marathon running, triathlon, stand-up paddleboarding, distance cycling, mountain biking, ice climbing, backcountry skiing, kayaking, and hiking. Athletes were either single-sport athletes, participating in and training for one discipline, or multi-sport athletes. Those that indicated more than one sport were training for and racing in more than one event, and/or using shorter distance events (marathon) and activities as training for ultra distance events. Participants were separated into three training groups by volume; *n* = 361 reported training less than 10 h per week, *n* = 140 reported training 10–20 h per week, and *n* = 26 reported training more than 20 h per week. *n* = 4 athletes provided non-quantifiable information about training and were excluded from correlational analyses related to ED risk and training.

Subjects mostly identified as “White or Caucasian” (494; 93.0%). 4 (0.8%) identified as “Black or African American”, 9 (1.7%) identified as “Asian or Pacific Islander”, 10 (1.9%) “Hispanic or Latino”, and 8 (1.5%) “Multiracial or Biracial”. 5 (0.9%) identified as “another ethnicity not listed”. Most respondents (*n* = 479 (90.2%) had participated in a UES event in the preceding twelve months and *n* = 508 (95.7%) planned on participating in a UES event in the twelve months after the survey. In total, *n* = 32 (6.0%) athletes reported having been diagnosed with ED prior to participation in this study per the “prior diagnosis” question in the sociodemographic section.

### 3.2 Overall ED risks per the EAT-26

Across the sample, the overall risk for ED, as measured by questions 1–26 of the EAT-26, was sizeable. Specifically, of the total participants, 11% (*n* = 58) reached at least the clinical threshold of 20 points on EAT-26, indicating the need for professional evaluation based on this instrumentation. For the behavioural questions in items A-E of the EAT-26, 20.3% of the sample (*n* = 107) exhibited at least one conspicuous behavioural answer (i.e., those that would require examination by a mental health professional per the EAT-26) (see Table 1).

**Table 1:** Frequencies of athletes with conspicuous scores on the behavioural questions of EAT-26.

Number of items with a conspicuous answer	Athletes	Percentage of athletes
0	424	79.7 (79.8)
1	78	14.8 (14.7)
2	22	4.2 (4.2)
3	7	1.3 (1.3)
4	0	0.0
Total	531	100.0

### 3.3 Effect of training volume on EAT-26 scores (adjusted and unadjusted analyses)

Table 2 presents the effects of training volume, gender, and age on EAT-26 scores. Training volume was significantly associated with ED (unadjusted: *F* = 31.494, *p* < 0.001), representing a stronger predictor than either gender or age. However, both gender and age contributed significantly as covariates, with females showing higher EAT-26 scores than males (*F* = 4.369, *p* = 0.037) and younger athletes scoring higher than older counterparts (*F* = 5.426, *p* = 0.020).

**Table 2:** Effects of training volume, gender, and age on EAT-26 scores.

Effect	Test/model	Statistic	*p*-value	Direction of effect
Training volume (overall)	ANCOVA/omnibus	*F* = 31.494	<0.001	Higher volume → higher EAT-26
Gender (female vs. male)	Covariate in model	*F* = 4.369	0.037	Females > males
Age (younger vs. older)	Covariate in model	*F* = 5.426	0.020	Younger > older

Higher scores indicate greater ED risk. Model adjusted for age and gender.

Tables 3, 4 summarizes EAT-26 scores across training-volume groups by gender and age. Mann–Whitney U tests indicated significant differences between groups when adjusting for age and gender, with athletes training more than 20 h per week reporting the highest EAT-26 scores (*M* = 16.42, *SD* = 15.57), followed by those training 14–20 h (*M* = 9.78, SD = 10.29), and those training <14 h (*M* = 6.42, *SD* = 6.95).

**Table 3:** EAT-26 scores by training-volume group and gender.

Training volume group	*N* (527) *Total/Female/Male*	Statistic Mean	SD
>20 h/week	26/15/11	16.42/18.73/13.27	15.57/17.51/12.59
14–20 h/week	140/64/76	9.78/11.38/8.43	10.29/11.61/8.87
<14 h/week	361/134/227	6.42/7.29/5.90	6.95/7.83/6.34

Pairwise Mann–Whitney *U*-tests for total: >20 vs. 14–20 (*p* = .024); 14–20 vs. <14 (*p* < .001); >20 vs. <14 (*p* = .004). Higher scores reflect greater ED risk; adjusted for gender.

**Table 4:** EAT-26 scores by training-volume group and age group.

Training volume group	*N* (527) *<36 y/36–45 y/>45 y*	Statistic Mean	SD
>20 h/week	11/8/7	16.36/16.50/16.43	18.35/13.82/15.06
14–20 h/week	43/59/38	11.44/10.10/7.39	12.05/10.24/7.68
<14 h/week	101/128/132	7.22/6.65/5.58	7.83/7.42/5.60

Higher scores reflect greater ED risk; adjusted for age.

**Figure 1:**
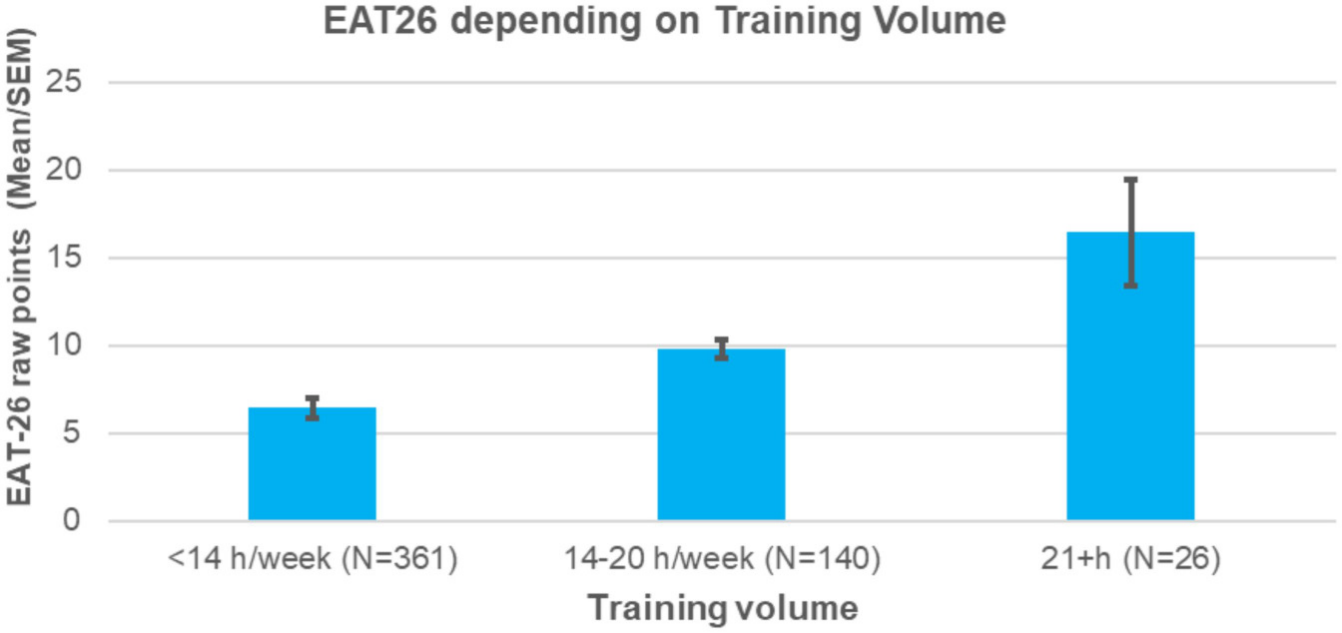
EAT-26 scores based on training volume groups.

These findings further support the association between higher training volumes and increased risk of disordered eating behaviours, as illustrated in Figure 1.

For the behavioural EAT-26 questions, training volume again contributed to ED risks (Table 5), with those in higher volume training groups exhibiting a great number of conspicuous behaviours.

**Table 5:** Conspicuous behavioural answers and training volume.

Item No. (*N* = 527)	Conspicuous answers						p_Chi_
	<14 h/week		14–20 h/week		21 + h/week		
	*N*	perc.	*N*	perc.	*N*	perc.	
A	32	8.9%	21	15.0%	5	19.2%	0.018
B	17	4.7%	11	7.9%	5	19.2%	0.004
C	14	3.9%	14	10.0%	7	26.9%	<0.001
D	5	1.4%	9	6.4%	3	11.5%	0.001

**Figure 2:**
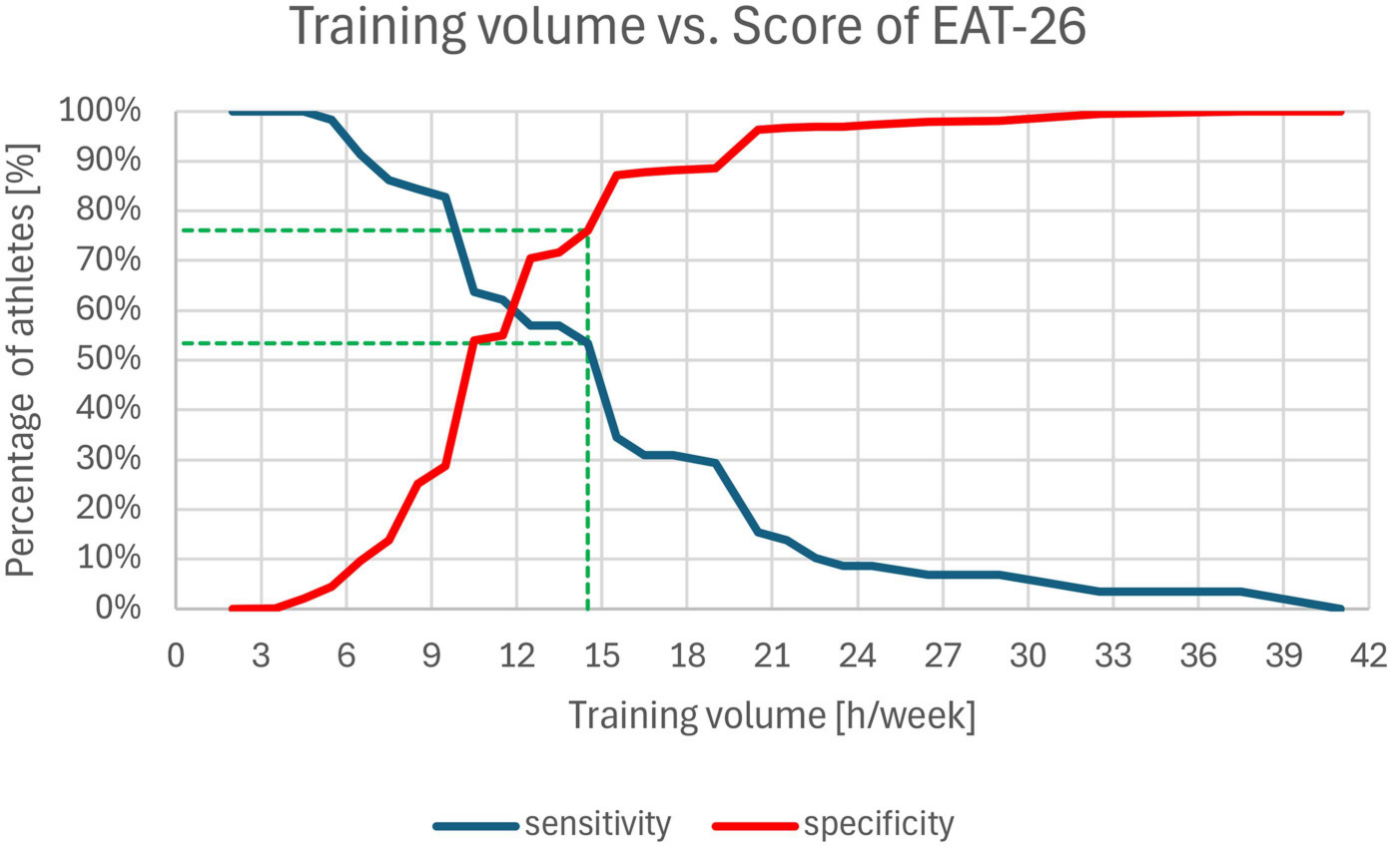
Predicting training volume threshold for increased ED risk in this sample.

Additional ROC analysis was used to determine the most selective threshold for training volume associated with heightened ED risk within this sample, maximizing the Youden Index [sensitivity [%] + specificity [%]−100%]. Notably, the analysis identified 14 h per week as the optimal threshold for predicting an elevated EAT-26 score (Youden Index = 29.5%) (“optimal” here referring to a balance that minimizes the exclusion of affected athletes as training volume increases). Sensitivity at this threshold was 53.4%, with a specificity of 76.1%, suggesting 14 h as the cut-off point for ED risk based on the above considerations (Figure 2).

## 4 Discussion

To the authors' knowledge, this is the first paper to compare training volume with risk for ED in UEA. Worldwide ED risk is said to be 7.8% (44) and prevalence rates in athletes are difficult to assess (45). In this context, our results revealed significant trends in ED risk in this sample, with 11% reaching the threshold on general EAT-26 scoring for follow-up by a mental health professional and 20.3% giving at least one conspicuous answer on the behavioural questions. This contrasts with 6.0% who had been diagnosed with ED prior to the study, thereby potentially suggesting an unmet care need in this population.

Additionally, training volume was a significant contributing factor to ED vulnerabilities, with those training above 14 h per week appearing to exhibit greater risks. This threshold aligns with findings from the ROC analysis, which identified 14 h as the point maximizing the Youden Index, balancing sensitivity (53.4%) and specificity (76.1%). Yet, this should not be interpreted as an absolute cutoff, as a substantial proportion of at-risk athletes may still fall below this threshold, while some above it may not exhibit ED symptoms. Rather, it serves as an indicator of heightened vulnerability, suggesting that increased training volume alone may contribute to elevated ED risk.

Concerningly, the overall nature of UES training may often require >14 h per week of training and anecdotal reports suggest that UEA recurrently exceed those levels (46). With this, the energy required to execute training protocols could leave athletes in chronic caloric debt which may affect physical and mental health outcomes. Generally, large amounts of physical activity have been linked with a declining sense of mental wellness, alongside increased ED risks (5, 17, 18). It is important to note that the question about training volume was designed to assess total number of sport training hours per week as well as any cross training and/or general exercise that athletes are likely to engage in (47, 48). This suggests that, the increased risk of ED >14 h is likely to be a concern for exercisers beyond the UES community. This is an important consideration as finding from this research could have implications for the general public that is prone to over exercise for perceived mental and/or physical health benefits.

A difficult challenge in UES may be maintaining energy balance though training and racing due to significant energy needs. UEA burn thousands of calories over the course of an endurance effort (i.e.,: more than 15,000 calories during a 100-mile ultramarathon) and caloric debt can become detrimental (49). The chronic inability to maintain energy balance may lead to the athlete being in a state of Low Energy Availability (LEA); this has been linked to somatic and psychiatric issues such as ED, overtraining syndrome, and hypothalamic amenorrhea (49).

A second phenomena associated with UES is the need to consume large amounts of food as calorie replenishment (24). Though it is improbable to fully mitigate such deficits (especially post-event), UEA must attempt to recover as much nutrition as possible irrespective of hunger or desire to eat (22, 24). That said, hunger will likely increase following the event, prompting the athlete to consume substantially more calories than usual (16, 17). Though this type of eating may not be considered pathologically as a binge, as the person would likely be lacking the feelings of guilt and the need to eat in secrecy, the effect of this kind of eating/need to eat is unknown. It is not clear whether the extreme hunger and quantities of food would engender distress, if an athlete would obsess about food, or if they may become ashamed of these behaviours.

Moreover, in recent years, the use of activity, nutrition, and fitness trackers has become more common among athletes, including UEA, with users numbering over 120 million on one platform alone (50). While these trackers can have positive effects, they have also been associated with detrimental mental health outcomes due to comparison, competition, obsessive fixation on data, and resulting poor body image (51, 52). Concerningly, the use of fitness trackers could possibly induce and exacerbate ED symptoms (53, 54). Notably, in our findings, the answer “I am aware of the calorie content of the foods that I eat” was one of the most commonly observed, which may be compounded by the use of tracking apps.

Within UEA culture, there is often a fixation on food and nutrition (2, 55). This may be due to a desire to optimize health and performance, but also conceivably due to a deeper fascination with the *need* to eat in a society that promotes dietary restraint, lean bodies, and hyperfitness (2). While the concept of food addiction is not formally recognized in the ICD-11 or DSM-5, research has identified patterns of compulsive eating behaviors in endurance athletes, highlighting the need to further explore their mental health implications (56). It is conceivable that ingestion of large amounts of food, whether due to metabolic demands or psychological factors, may lead to compensatory exercise in certain athletes, creating a transactional relationship between food and physical activity that may cause significant psychological distress (56); though this was not identifiable within the current study design.

In response to the complexities of maintaining energy balance, professional teams have increasingly integrated nutritionists as part of a structured support system (57). Initially observed in elite-level endurance sports, this trend is now extending into amateur competitions as training practices become increasingly professionalized. Though such guidance may reduce the likelihood of athletes engaging in self-directed nutrition management strategies, inadequate knowledge may affect dietary intake in UES (58). Equally, when nutritional intake is frequently monitored and structured, it may inadvertently introduce psychological stress, particularly for athletes vulnerable to disordered eating and weight and body image concerns (59, 60).

Additionally, certain diet-focused individuals may be drawn to UES for intentional weight loss (55). Though UEA also participate for personal satisfaction, general health, and social connection, and other reasons, weight and body concerns have been identified (61). Again, in our results, respondents most frequently answered “always” to the following statements: “I am terrified about being overweight”, “I am aware of the calorie content of the foods that I eat”, “I am preoccupied with a desire to be thinner”, and “I think about burning up calories while I exercise”. Dieting is associated with risk of ED with or without engaging in physical activity (62). Interestingly, individuals may be able to lose weight while training for UES without cutting calories as long as physical activity remains high. This could encourage UES participation for some athletes and, anecdotally, may cause additional distress from the cyclical need to add physical activity in response to dietary intake (63).

Moreover, it is unknown whether the abnormal nutritional needs of UES could be triggering and therefore harmful to a person with ED (i.e., if a person with anorexia would be drawn to the outsize caloric burn of UE sport to give themselves permission to eat, if a person with binge eating disorder might hide their behavior under the guise of “replenishment”, or if a person with bulimia may be compelled to shift to purging through physical activity). Though these behaviors would be concerning, it is perhaps more concerning to consider what would happen if these athletes were to stop training and whether they have support systems in place to manage an active ED. As an additional consideration, though EXD is not yet recognized by either the DSM-V or the ICD-11 as classified diagnosis, it can contribute to an athlete's risk for ED. UEA have been found to be at risk for EXD and may influence certain UEA to continually add volume, avoid periodization of training, and be reluctant to take rest days (64, 65).

Another cultural feature of UEA may be the attempt to normalize certain characteristics and a willingness to self-identify with terminology related to mental illness i.e.,: “crazy”, “madness”, “weird”, etc. (2). UES is often described as a sport for “the crazies” and anecdotally, UEA have described their training habits as “insane” (66, 67). The use of these terms may be another attempt to normalize abnormal behaviors, but likely both increases stigma and impedes UEA from seeking mental health care (68).

Aside from logistical considerations of UES and the unique cultural characteristics of the community, personal characteristics of athletes could also contribute to ED vulnerabilities. Research into personality traits and ED discusses an association between neuroticism and ED, and a similar correlation between neuroticism and UEA with ED has been found (29, 69). Those who experience ED often have a higher tolerance for pain, a characteristic also found in UEA (70, 71). UEA have been found to score higher on assessments of grit, and grit may contribute to ED risk when it supports the use of physical activity as maladaptive compensation for mental disorder (72, 73). Mental toughness is a construct that may play a role in the development of ED in athletes and has been shown to be a significant factor in UES (74, 75).

Finally, it is important to consider whether the potential for mental disorder in UEA is a function of the physiological implications of stress caused by high volume training. As the Hypothalamic Pituitary Adrenal (HPA) Axis is integral in management of the body's stress response, it can become dysregulated with the continual training–particularly when training not accompanied by adequate recovery which can be characteristic of high volume training (76, 77). UES have connections with HPA axis dysfunction, inflammatory responses, and immune system suppression (63, 78, 79). HPA axis dysfunction, which has been regarded as the cause of mental disorder as the stress hormone cortisol negatively impacts brain function (80). The link between the HPA axis and ED has been discussed as hormonal response from both biological and environmental factors may influence both the onset and development of ED (81). This suggests that stress, whether chronic or traumatic, which activates the HPA axis may potentially precipitate disordered eating due to complex alterations in neuroendocrine function (81). Thus, theoretically, there may be a danger in the relationship between chronic stress and ED, as under-eating due to the chronic stress from training may cause additional physiological stresses related to malnutrition (81).

## 5 Strengths, limitations, and directions for future research

This analysis on a sample of UEA explored training volumes and ED risk using validated psychometric tools, showing that risk may be positively related to training volume and those engaging in physical activity >14 h weekly may be at higher risk for ED. Strengths of the research included a robust sample size, which is higher than other studies on ED in UES (11). Furthermore, that a wide range of ages (19–73) were captured in the study and the gender split (59% male, 41% female) was more balanced compared to recent demographic studies on participation trends (1, 82).

Nevertheless, the research does have limitations that may limit its wider generalisability, especially given its cross-sectional design. First, we are unable to capture an understanding of the intensity of the athletes' training hours; different activities would represent different intensities (i.e: yoga vs. a heavy strength-training session), but the aim of this work was to simply capture the number of hours that participants were engaging in physical activity, generally. Though attempts were made to include as large a sample as possible, it was still limited to those athletes who learned of the study through advertisements, by word-of-mouth, or because a prominent athlete may have promoted the study via social media. With this, the participants would likely have been limited to those who had access to digital media. That said, social media is often a prominent platform for UES and has been portrayed as both as a source of knowledge sharing and community engagement (2, 83). Additionally, the research was based on self-report data, particularly regarding participation in UES and training levels, which can carry a certain amount of bias (84), and was gathered during the COVID-19 pandemic; at this time, is possible that athletes' training volume may have changed, though the study questions were designed to capture habits prior to this.

As previously stated, efforts were made to recruit a large number of athletes to participate in this study through online platforms specific to UEA. However, the resulting subject pool was not racially diverse and it is unclear if results would be different in a more diverse population, which could be scrutinised in future research. Likewise, there is a lack of scientific inquiry into personality characteristics and traits that may compel an athlete to push for more physical activity in the context of UES. In general, it is crucial to understand why certain athletes train at high volumes for extended periods of time. Currently, it is unclear whether perceived benefits may be physical, mental, or a combination. Therefore, investigations should attempt to discover what characteristics are shared by UEA and how these relate to characteristics that overlap with ED risk.

Whilst this research focussed on UEA, other studies should examine the correlation between training volume and ED risks in populations of exercisers who do not participate in UES. This would allow for a clearer understanding of whether the findings we identified were solely related to UES, UEA, or a function of the overall physiological implications of high physiological stress through physical activity. In addition, follow-up studies on the synergistic effects of high volume training and ED risk would be potentially helpful in comprehending how fluctuations in training volume in a particular individual may affect relative risk. The reasons why ED risk increases at 14 h of physical activity per week specifically are not discernible from this study. However, this threshold may represent a need to investigate the relative healthfulness of large volumes of physical activity and the potential a U-shaped relationship between health and exercise, generally.

## 6 Conclusion

This study showed a significant association between high-volume training and an increased risk of eating disorders in ultra-endurance athletes, with a higher risk at high-volume training of more than 14 h per week. Although participation in UES itself may not be harmful, the results indicate the need for caution when planning training and a better understanding of the psychological and physiological consequences of high volume training on athletes. Given the lack of previous research in this area, the findings of this study open the way for further scientific analysis, especially through longitudinal studies and a wider population of athletes, but also highly active recreational athletes. As there are variations in risk among the total population of participants, it is also clear that there is a need for more sensitive, individualised screening and treatment protocols.

## 7 Implications and recommendations

- Increasing hours of exercise per week may not contribute to increasing mental health benefits and may actually be physiologically and psychologically harmful.
- More nuanced messaging around general exercise guidelines should be employed, generally. This may be especially important for those beginning an exercise regime who may incorrectly assume that moderate amounts of physical activity would not be sufficient to gain benefits.
- Athletes should be made aware of the potential risks of UES (or potentially, of engaging in high volumes of physical activity generally) given that it remains unknown whether negative outcomes may be due to UES participation or the characteristics of those who are drawn to participate in these sports.
- Healthcare practitioners should prioritize enquiring about the amount of physical activity/training/exercise a patient is engaging in, as well as be prepared to modify diagnostic and treatment considerations accordingly.
- Athletes should be educated about eating behaviors that would align with disorder-based classifications, generally, as greater awareness may impede the development of such issues.
- Clinically, healthcare practitioners should consider that the typical signs and symptoms of ED could be masked by the unique habits, nutritional needs, and training protocols of UES.
- Sport-informed, registered dietitians should be prepared to advise athletes on how to address the challenges of adequate refueling when training for/competing in ultra endurance events while remaining within guidelines of safe/healthful eating practices.

## Data Availability

The raw data supporting the conclusions of this article will be made available by the authors, without undue reservation.
